# A Review of IoT Sensing Applications and Challenges Using RFID and Wireless Sensor Networks

**DOI:** 10.3390/s20092495

**Published:** 2020-04-28

**Authors:** Hugo Landaluce, Laura Arjona, Asier Perallos, Francisco Falcone, Ignacio Angulo, Florian Muralter

**Affiliations:** 1 DeustoTech, University of Deusto, 48940 Bilbao, Spain; perallos@deusto.es (A.P.); ignacio.angulo@deusto.es (I.A.); florian.muralter@deusto.es (F.M.); 2 Paul G. Allen Center for Computer Science and Engineering, University of Washington, Seattle, WA 98195, USA; arjonal@cs.washington.edu; 3 Electrical and Electronic Engineering Department, Public University of Navarre, 31006 Pamplona, Spain; francisco.falcone@unavarra.es

**Keywords:** passive sensors, computational RFID, sensor tags, RFID sensor network, wearable RFID, energy-harvesting, standards and communication protocols, wireless communications

## Abstract

Radio frequency identification (RFID) and wireless sensors networks (WSNs) are two fundamental pillars that enable the Internet of Things (IoT). RFID systems are able to identify and track devices, whilst WSNs cooperate to gather and provide information from interconnected sensors. This involves challenges, for example, in transforming RFID systems with identification capabilities into sensing and computational platforms, as well as considering them as architectures of wirelessly connected sensing tags. This, together with the latest advances in WSNs and with the integration of both technologies, has resulted in the opportunity to develop novel IoT applications. This paper presents a review of these two technologies and the obstacles and challenges that need to be overcome. Some of these challenges are the efficiency of the energy harvesting, communication interference, fault tolerance, higher capacities to handling data processing, cost feasibility, and an appropriate integration of these factors. Additionally, two emerging trends in IoT are reviewed: the combination of RFID and WSNs in order to exploit their advantages and complement their limitations, and wearable sensors, which enable new promising IoT applications.

## 1. Introduction

The paradigm of the Internet of things (IoT) is mainly focused on providing thousands of small interconnected devices that can collaboratively work together with a common purpose. The increasing number of these small interconnected devices is enabling the IoT to become a reality. These devices are smart but simple objects with sensing and wireless communication capabilities [1,2]. In this framework, two technologies are mainly used, and are becoming the two fundamental pillars of the IoT: radio frequency identification (RFID) and wireless sensors networks (WSNs) [3]. Both technologies are focused on wireless sensing and communication, which are the two main needs of the IoT.

RFID is an auto identification technology that uses two main types of devices: a reader, which is the master of the communication, and the tags, which have an associated electronic code they use so as to be uniquely identified. The reader interrogates these tags using radio frequency (RF) signals, and the tags respond with their identification code (ID). Tags may also incorporate a sensor, in which case they will also backscatter the data from their sensor. Tags can be active (powered by a battery) or passive (harvesting the energy from the reader’s RF signal). RFID is a consolidated technology for the identification of assets, security, and track-and-trace applications with a high and increasing density of tags available within the interrogation zone, particularly using passive tags [4].

WSNs are collections of nodes implementing sensors that collect data in a distributed manner and wirelessly transmit it to a main node. A WSN is mainly composed of sensing nodes, gateways (base station or router), a coordinator, and a PC server. The sensing nodes collect the information from their respective sensors and communicate with a PC server through the gateways. WSNs are widely used in medical, environmental, military, and security applications [3].

Whilst RFID is used to identify and track devices, WSNs collaboratively gather and provide information from their sensors. These two technologies can work together to exploit their advantages and complement their limitations [5,6]. Challenges that arise in this regard include the transformation of RFID systems with identification capabilities into sensing and computational platforms, as well as determining the appropriate architectures for wirelessly connected networks of sensors. This means that RFID can be used as a WSN itself, constituting a network of sensing nodes, connected to a PC through the coordinator/reader; or it can be integrated into a different WSN, extending the capabilities of both systems. Thus, the combination of RFID and WSN represents a very promising approach to meet the current challenges in IoT, and has created an opportunity to develop novel IoT applications [7,8,9,10,11]. Moreover, another outstanding type of wireless sensors are wearable sensors. These can also be either passive or active, and they can be RFID tags or sensors belonging to a WSN. All of this represents a hot research topic, and is reviewed in this paper.

The rest of this paper is organized as follows. Section 2 reviews RFID sensing technology and Section 3 reviews WSNs. Section 4 analyses two of the main trends in current IoT research: the integration of RFID with WSN, and wearable sensors. Section 5 concludes this review paper.

## 2. RFID Sensing Technology

The last decades have witnessed a rapid growth of RFID technology for identification and tracking applications. The ability of RFID to identify, trace, and track information using easily deployable tags is now enabling applications beyond its traditional use in supply chain management: it is now employed in new areas of sensing, actuation, and even user interaction. RFID tags can be used as standalone sensors as well as RF front-ends for other off-the-shelf commercially available sensors. Enabling the sensing ability into RFID technology can make the system gather information from real-world objects and seamlessly integrate it within the IoT. There are two main types of RFID tag-based sensing systems:

- **Analog RFID sensing**: These systems perform an analog processing of the physical signals related to the communication between the reader and the tag, with no dedicated sensing electronics. The reader is able to obtain much more information about the target, more than just identification, without the need for additional electronics. Analog RFID sensing relies on the knowledge that the performance of an RFID tag is affected by the hosting object, and hence it is possible to retrieve sensing data simply by evaluating the variation of the signals backscattered from the tags. Sensitive coating materials or lumped components displaced over the antenna are also used to achieve a more specific response of the device [12].
- **Digital RFID sensing**: Tags are integrated with electronic components, such as sensory material, analog-to-digital converters, and a microcrontroller, to make an integrated sensor module [13]. These systems are referred to as Computational RFID (CRFID). CRFID systems permit running programs on embedded computers using only scavenged Radio Frequency (RF) energy. Battery free, “invisible” sensing and computation is key to truly ubiquitous computing applications for the IoT. The CRFID tag is used as a communication interface for transmitting data. Passive RFID sensors harvest the RF energy from RF radiation to power the circuit, perform the sensing task, and save the data in the RFID chip to be accessed by RFID readers.

Both analog and digital RFID sensing can provide a variety of sensing capabilities to IoT systems. The decision to use one or the other will depend on the particular application. Analog RFID sensing systems generally have a lower cost, but, compared to digital systems, involve a critical trade-off between sensing and communication requirements. Accordingly, the dynamic range and the resolution of the measurement signals are limited by the minimum required interrogation distance and by the unavoidable uncertainties arising from the propagation of an electromagnetic wave in a practically random environment. Also, they are typically much less accurate than a digital CRFID. CRFID systems are practically immune to environmental interactions (data coming from the specific sensors are digitally encoded and transmitted through a regular RFID interrogation), and hence they can produce more accurate and very selective outcomes at the expense of a higher level of harvested energy.

### 2.1. Challenges

**Figure 1:**
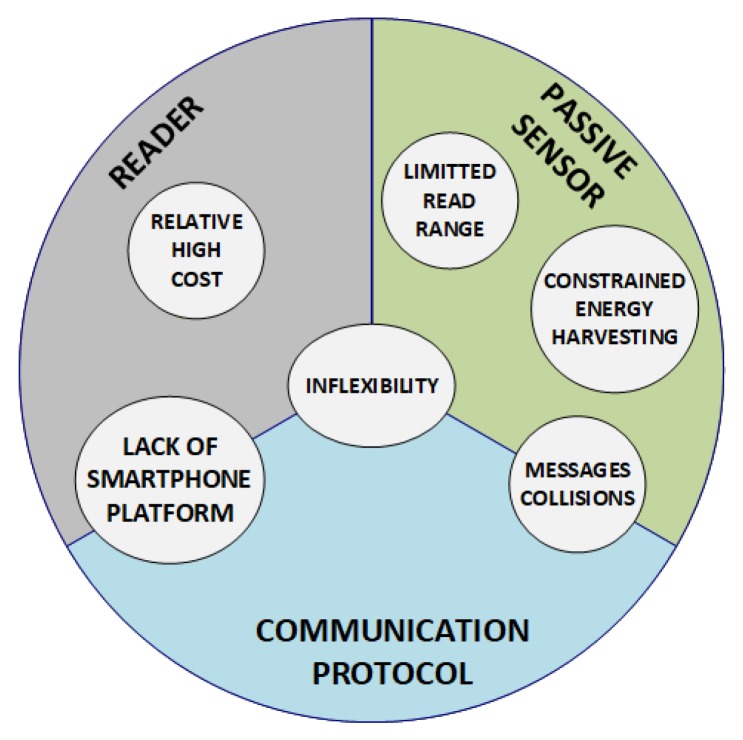
Overview of the main challenges that prevent radio frequency identification (RFID) from becoming pervasive. The challenges are classified according to the RFID component or components (reader, passive sensor, communication protocol) which they affect.

Wireless RFID sensors (both digital and analog) have not yet become pervasive in the home and personal electronics market, with the advantages of RFID for the purpose of tracking and managing everyday objects readily apparent. There are several main obstacles that prevent RFID sensing from becoming pervasive. This review identifies 6 main challenges. Figure 1 shows an overview of the main challenges of RFID, which are classified according to the RFID component or components (reader, passive sensor, communication protocol) which they affect.

- **Limited energy harvester and read range**: These are considered to be two of the most important limitations because both sensor nodes and RFID tags are made of scarce resources [14,15]. Existing RFID platforms implemented in the IoT are mostly passive, that is, they cannot operate or sense data without being placed inside the reader’s reading zone. The integrated circuit (IC), the microcontroller unit, and the sensing module on a passive tag are powered by harvesting the RF energy transmitted by the reader, and communicate by backscattering the incident signal. This implementation reduces the manufacturing cost by keeping IC costs low. However, the long-range communication and power hungry sensing capabilities will be limited by the power available at the tag.
  Furthermore, the maximum power transmitted by the reader is constrained by the Federal Communications Commission (FCC) (or similar regional organization) at 1 W (30 dBm), assuming an antenna with a maximum gain of 6 dBi. Only a fraction of this transmitted RF power is received at the IC after path losses and polarization mismatches.
  Although all the components are typically designed to be power efficient, the operation of the logic of the sensors is more complex and time consuming. Therefore, it is still a challenge to power all the components and cover the operations of the logic with only harvested RF energy. This challenge is more notable when the sensors are implanted in the materials under test, because the RF signal is attenuated by the surrounding materials and the received RF energy can hardly power all the operations, which seriously affects the read/write range of the RFID sensor.
- **Sensor responses collisions**: An RFID sensing application is composed of at least one reader and several RFID sensors, which include at least one sensor. The tag collision problem acquires the main focus when interrogating these tags. The communication channel is shared among them and, therefore, their responses need to be arbitrated in order to avoid simultaneous responses that will lead to collisions. This problem is one of the main causes of energy wastage, increases in identification time, and decreases in the read rate [16]. Due to the current increasing number of sensor tags in a shared reader interrogation area, this problem is the subject of increasing concern. Efficient anti-collision protocols for streaming sensor data are needed to minimize the impact of collisions.
- **Lack of flexibility**: Current sensor RFID tags usually come with a single sensor or, in some cases, with multiple built-in sensors. But once they are manufactured, they cannot be replaced or reconfigured without a costly redesign and reproduction. Since the IoT is an open, dynamic, and versatile global networking and sensing system, the generality, modularity, and reconfigurability of the sensing nodes/platform are essential for the their adoption in the future IoT architecture. Furthermore, commercial RFID readers are generally black box systems that only allow limited configuration [16] and are only capable of implementing the current UHF RFID communication standard named EPCglobal Class 1 Generation 2 (EPC C1G2) (ISO/IEC 18000-63). Thus, it is not possible to implement new communication protocols beyond the EPC C1G2 that meet the demands of novel and emerging RFID-based sensors. Although there are some publications in the literature that propose a flexible RFID reader based on a software-defined radio, there is a lot of room for improvement in this regard [17].
  Another limitation is related to the current lack of an UHF RFID mobile sensing platform. Currently are portable commercial RFID readers, which do not need to be plugged in to operate, but there is a lack of a smartphone-based platform that enables the use of unmodified smartphones to read data from UHF RFID sensors. It would be very beneficial and practical to be able to read RFID sensor tags by using a common smartphone with some additional hardware components (such as an UHF antenna).
- **Cost**: The cost of commercial RFID readers is relatively high compared to the cost of sensors and tags.

### 2.2. Applications

The use of RFID technology for sensing our physical world has expanded tremendously in the last decade. New applications, using both analog and digital sensing, continue to emerge, and they will revolutionize the emerging IoT applications in a wide variety of areas.

RFID sensors are revolutionizing the healthcare industry in ways that benefit both the patient and medical providers [18,19,20]. Wearable and wireless devices allow efficient and continuous medical monitoring. Wireless sensors do not limit the patient’s movements, thereby improving their quality of life. Similarly, medical providers can gather data, facilitate inter-departmental communication, and dispatch emergency care more efficiently.

The agriculture of the future will demand efficient solutions by means of technology and sensors [21,22]. Several studies have demonstrated the need to significantly increase the world’s food production by 2050. The key to sustainable food production lies in the thoughtful and purposeful use of technology and sensors. Innovations such as moisture sensors, smart irrigation, drones, as well as self-driving and GPS-enabled tractors, have helped farmers in recent years. RFID provides an energy-efficient solution to monitor key parameters such as humidity, temperature, moisture, and light strength.

Regarding transportation, smart roads and infrastructure, self-driving and environment-connected vehicles, and people-centric integrated transportation applications, are just a few of the innovations in the transportation industry built upon the technology of RFID sensors. Current examples of RFID sensors in transportation system include References [23,24,25].

RFID technology is already being widely adopted across the retail sector [26,27]. RFID tags can identify every product in a store with a unique identifying number; they reduce the need for human resources, and eradicate human error by automating processes; they enable simultaneous product scanning; offer real-time stock information; provide new ways of advertising; and increase the security of the staff, equipment, and stock. Thus, adding sensors in those tags can provide additional features, such as information about the room temperature, moisture, and product degradation.

RFID indoor positioning (IP Systems) has been selected as one of the indoor wireless location technologies, and there has been a rapid development [28]. RFID technology has several advantages for positioning, highlighting very low cost, lightweight, and multiple tag identification. One of the most famous RFID positioning algorithms is LANDMARC [29]. The LANDMARC system is a typical reference-tag-based RFID positioning method. By introducing a reference label to assist positioning, a reference label matrix is arranged in the positioning space; Received Signal Strength Indication (RSSI) values of the unknown label and the reference label are measured; and the nearest neighbor K value algorithm is used for positioning.

RFID sensing also presents promising applications for industry [30,31,32]. The relatively low cost and maintenance of RFID sensor tags, their versatility in use and application, and the possibility of exploiting conventional RFID readers and standard protocols, are expected to allow the massive integration of RFID platforms in industrial scenarios, from automotive to hot/cold manufacturing, from logistics to predictive maintenance. By embedding RFID sensors directly in the products, it is possible to generate smart-objects able to remotely interact with the surrounding environment over their entire life-time, from manufacturing and testing, in-exercise, up to removal. Real-time monitoring of industrial platforms and processes by means of RFID passive technology can be considered a valid and applicable instrument. Thanks to new-generation sensing oriented ICs, it is nowadays possible to deploy complex wireless sensors directly within objects in manufacturing or over equipment and machinery.

**Figure 2:**
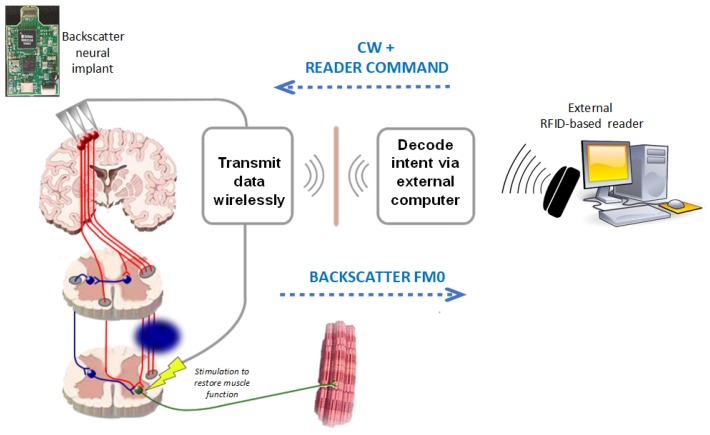
Communications scheme using a sensing RFID neural implant [33,34].

Finally, RFID sensing is emerging in the field of neuro-engineering (see Figure 2). Implantable biomedical devices are certainly one of the hottest application areas of RFID because of the great potential that wireless power and data communication capabilities, inherent in RFID, bring to this field. Hence, the RFID physical layer is ideal for applications such as neural recording, where implanted sensors do not require any source of energy of their own except for an external RF field [33,34]. An overview of the main areas of application of RFID sensing are shown in Table 1.

## 3. Wireless Sensor Networks

A WSN consists of a collection of spatially distributed and independent devices that collect information and digitally transmit it over a wireless channel. A WSN can use hundreds of sensors, accompanied by gateways and a coordinating device, to sense the environmental or physical conditions of a system, and to monitor or control it. Each node contains one or more sensors, which can be passive or active. These sensors communicate with each other to transmit the information to a server PC that manages the information of the entire network [35]. Typical technologies and communication standards used in WSN are WiFi and Bluetooth on the physical and Media Access Control (MAC) layers, and ZigBee and 6LowPan protocols in the network, security, and application layers. A compilation of the most common technologies found in IoT applications is shown in Table 2. However, there are other promising technologies that are increasingly being used such as LoRa, IEEE 802.11ah and NB-IoT for single-hop networks, WirelessHART, ISA100.11a and BLE for multi-hop networks [36].

### 3.1. Challenges

The introduction of wireless communications into different types of applications can be a difficult task due to their heterogeneous requirements. This fact indeed affects IoT applications, since they interconnect objects in different environments, such as cities, industry, or logistics, and each environment has its own requirements. Nevertheless, all WSNs, regardless of their application, present several common challenges. Next, the main challenges will be reviewed.

- **Reliability**: Nodes must be available at any time to monitor critical areas, but this can lead to unnecessary communications or computations, which can cause the batteries of the nodes to run out of charge. Also, time synchronization between the nodes and spectrum sharing techniques are of the utmost importance to ensure the integrity of the data or avoid uncertainty in the sensed data, without consuming all the radio resources [37].
- **Energy consumption**, including harvesting, conservation and usage. Nodes must be power efficient and must be capable of low power communication with low cost on-node processing. Whether the nodes use a battery or harvest energy from the surroundings, their power consumption must be low in order to maximize their efficiency. The optimal node would be able to harvest energy from its surroundings and not waste any of that energy in its operations [38].
- **Scalability**: IoT solutions will involve thousands of smart devices, and this number will be dramatically increased in the coming years. Hence, a WSN must be sufficiently scalable so as to be able to integrate new nodes and provide (Quality of Service) QoS services involving heterogeneous devices, working for long periods [39].
- **Communication mechanisms and protocols**: There are 4 types of MAC communication strategies: Fixed, On-demand, Random, and Hybrid assignment [36]. Fixed assignment protocols divide the resources between the nodes in predefined time slots; On-demand protocols provide resources to each node on demand; Random Assignment protocols randomly divide the resources; and Hybrid Assignment protocols combine the previous three strategies. The Fixed assignment strategy adds determinism at the expense of an inefficient use of time; whilst the On-demand assignment lacks determinism and is not suitable for industrial real-time applications. In between these strategies are the Hybrid assignment strategies, combining fixed and random assignments. Moreover, the WSNs used in the IoT need to implement Internet functionalities. The most used one is the Internet Protocol (IP), to allow the routing of the messages among the wireless network.

**Figure 3:**
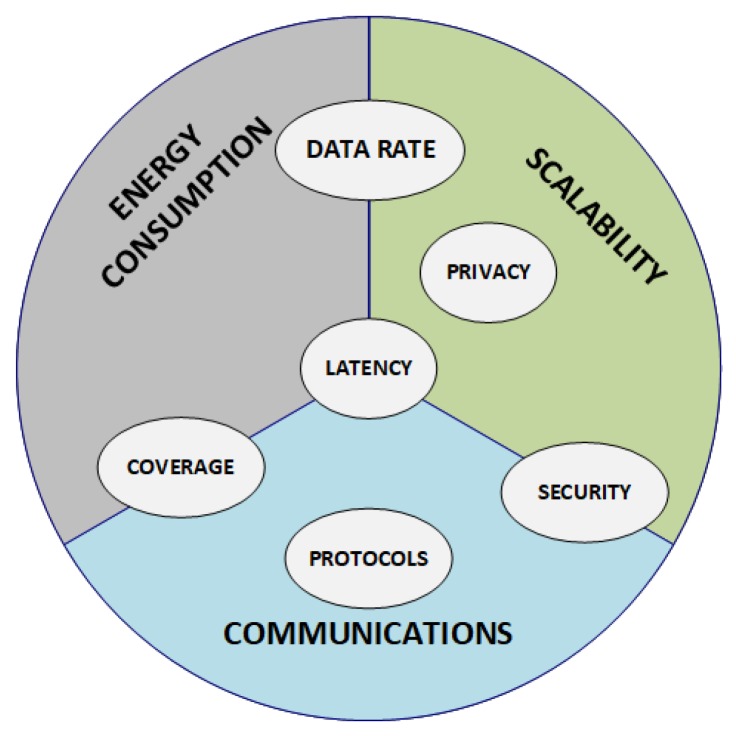
Overview of the main challenges facing WSN technology. The most significant challenges contain other related ones.

Figure 3 shows the main identified challenges of WSNs, explained above, and additional challenges that can affect these main ones. It can be seen how latency is affected by the communication mechanisms, such as coding techniques or the routing and rerouting of the messages, as well as the scalability of the system; and at the same time it degrades the energy consumption of the network. Also, the data rate balances the energy consumed by the system and the scalability to interrogate a higher number of nodes in the same amount of time. The data rate is directly related to the bandwidth: working in GHz channels will ensure higher data rates than those of the licensed wireless technologies (400 MHz, 800–900 MHz), because the latter use narrower channels than the ones in the GHz band. Additionally, coverage can affect the scalability of the system, since more nodes would be needed. But not only coverage affects it but also, the power of the device, the radio regulations, the coding, the modulation, the properties of the radio propagation, and the topology of the network. A network with a lower data rate covers a wider area at the expense of an increase in latency [39]. Moreover, security and privacy are a really important challenge that affects all IoT technologies, and thus these must also be ensured in these networks. This involves data confidentiality, avoiding data sniffing; data authenticity, avoiding packet injection with misleading information; and data integrity, taking into consideration that transmission errors are inherent in a WSN, the integrity of the transmitted data must be ensured [40].

### 3.2. Applications

WSNs are the key to enabling IoT applications [41]. Currently, there are several types of sensor nodes that can enable a great variety of applications: Terrestrial sensors, which are deployed or pre-planned in terrestrial environment; Underground sensors that can be deployed in caves, mines or under the soil, something which limits the replacement of their batteries; Mobile sensors, which can monitor the physical environment, the habitat or track a target; and multimedia sensors that can store, save or process multimedia data such as audio or video, requiring high bandwidth, QoS and power.

Considering all these possibilities, WSNs have revolutionized and improved fields such as ecology, transport, entertainment, health, and security among others. In particular, in road transport, WSNs are used to gather data to gain an insight and provide services to manage traffic. This is being implemented using novel architectures based on edge and fog schemes [42].

Indoor positioning is also another field which is being highly benefited from the use of WSN. The proposed solutions in the literature seek for a balanced system between communication complexity, storage scale, localization error and location accuracy [43]. These distributed solutions use sensor nodes to localize using various localization techniques, the most popular categories are range-based and non range-based algorithms. Among the first category techniques, RSSI with Extended Kalman Filter (EKF) [44], the Angle of Arrival (AoA) [45] and the Time of arrival (ToA) [46] are some of the most relevant ones. For the second category, also called range-free localization algorithms, the types of techniques are Distance Vector Hop [47], Hop Terrain [48], Centroid System [49], the Approximate Point in Triangulation [50] and the Gradient Algorithm [51]. ZigBee or Ultra Wide Band (UWB) nodes, are cost-effective, consume low-power, support low data rates and provide advantages such as security, robustness, and reliability. Using RSSI and Link Quality Indicators (LQI) from the network nodes, and using sensor fusion techniques, the position and movements of people can also be calculated by step length estimation [52].

Related to a smart environment, a solution is presented to ‘self detect’ earthquakes, tsunamis or to locate potential survivors by setting up sensors along the extensive coastal stretches [53]. Indoor positioning is Another typical application for a WSN is the monitoring of the air pollution in cities [54]. Smart homes are another field of application, so as to decrease the impact of our homes on the environment, a green house manager with resource management and an anti-theft system is presented in Reference [55]. Several solutions can also be found in the field of agriculture [56]. To improve these processes, data is acquired using a WSN and processed by machine learning techniques [57].

## 4. IoT Promising Technologies

The previous sections have shown why RFID and WSN are two fundamental pillars that will enable new promising IoT applications [3]. This section presents two technological trends for new IoT applications. The first one is the integration among the two identified technologies in the previous sections, RFID and WSN; and the second one is the wearable feature for, at least, these two technologies to propose new IoT applications.

### 4.1. RFID and WSN Integration

RFID networks are focused on detecting the presence of tagged objects, while WSNs are used for sensing the environment and positioning objects or people. On the one hand, RFID has several features, such as the ability to identify and track objects, and its ability to harvest energy from radio frequency signals, that can improve some of the drawbacks of a WSN. On the other hand, the integration of RFID with WSN enables the RFID technology to increase the read range and be part of a highly intercommunicated network with a more popular protocol, such as IP. This protocol cannot easily be implemented in very simple nodes such as RFID tags; thus, the integration between RFID and WSN facilitates the connection of tags to the internet by providing them the mechanisms to integrate their IDs in the IP protocol. This is done adding a routing function to the RFID reader, so that it can forward information to or from other readers, and to increase the reading distance from several meters to 100–200 m.

An integrated WSN node is composed by a coordinator micro-controller, an RFID reader, and an RF transceiver. This micro-controller manages the reader and the rest of the components of the node. And an integrated RFID tag with WSN capabilities, is composed of a micro-controller, a sensor and the RF front-end to communicate with other nodes or tags. These tags not only transmit their ID, but also, data from their attached sensor.

An RFID passive tag obtains energy from the readers signal, as opposed to a battery like WSN nodes. The main research contributions on the integration of RFID and WSN are focused on the energy efficiency of the integrated system, by trying to take advantage of the low power consumption of the RFID tags whilst achieving all the communication capabilities of WSN nodes. To seek this optimization, several magnitudes such as energy conservation, recharging and optimization of the harvested energy need to be taken into consideration. This is still a pending task to be optimized in order to achieve a proper energy efficient integrated system [58]. A summary of RFID, WSN and the combination of both is shown in Table 3.

**Figure 4:**
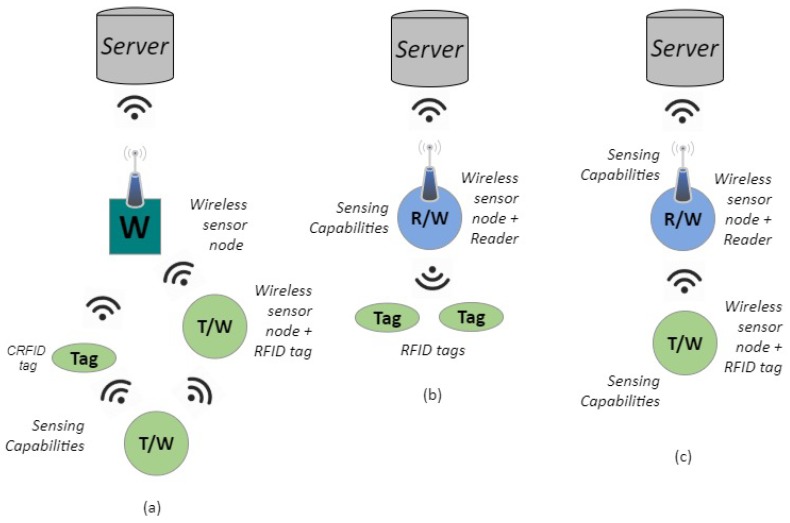
Types of integration in WSN and RFID: (**a**) at tag level, (**b**) at reader level, (**c**) hybrid.

There are three levels of integration among RFID and WSN producing three different architectures [3,59]—RFID tags with WSN nodes, RFID readers with WSN nodes, and a hybrid integration (see also Figure 4).

- The category of RFID tags with WSN nodes involves the possibility that the end node can be an RFID tag or a WSN node (see Figure 4a), and thus, there are two cases: integrated sensors with limited communication capabilities for the first case, and integrated sensors with extended communication capabilities for the second [58].
- For the category of RFID readers with WSN nodes (see Figure 4b), the integration is at the reader level. This increases the capabilities of the integrated system, allowing the identification of a tag, through the WSN node, at a very long distance, at the expense of an increase in the WSN node’s power consumption [8].
- The last category, hybrid integration, is a mixture of the previous two (see Figure 4c). An RFID reader is attached to a WSN gateway and thus, RFID tags coexist with WSN nodes as sensors. This Reader/Gateway collects data from either RFID sensors or sensor nodes and forwards the data to the coordinator of the network. Figure 4 shows a diagram of an example of hybrid integration.

The integration of RFID and WSN is an inexorable next step in IoT research to evolve the technology to a higher level. But first, there are several challenges that must be faced. The energy management of the integrated network is one of the most important issues to take into account. Also, the arbitration of nodes responses to avoid collisions is another important challenge in order to produce an efficient network, reducing interference in high density environments. Also multi-hop must be considered to avoid reader to reader interference and to maximize the read range of the passive side of the network. Moreover, in order to produce affordable systems, the manufacturing processes must be cost effective. As it can be inferred, the integration process of these two technologies is in a premature state, with several drawback to be solved yet such as, energy conservation, real-time dynamic performance, semantic data cleaning, data filtering, localization of nodes, and security concerns [58,60].

Regarding the existing applications, one of the main ones integrating RFID and WSN can be found in managing a supply chain. Retail is a typical field where RFID fits perfectly. This system is improved by being provided with IP wireless connectivity, increasing the connectivity capabilities, communication range, GPS and artificial intelligence techniques to process the acquired data, providing services to improve product tracking, improve delivery routes, and avoid shoplifting [61].

### 4.2. Wearable Sensors

One of the key areas of development within the framework of context aware environments and the progressive adoption of the IoT is related to human–user interactions. In this framework, wearable sensing technology has recently and rapidly moved from largely a vision of science fiction, to a wide array of established consumer and medical products. Wearable sensors have recently seen a large increase in both research and commercialization. However, developments in wearable sensors have been a mix of both progress and setbacks.

In human–user interactions, wireless body area networks (WBAN) and wireless personal area networks (WPAN) play a key role in providing specific communication abilities in order to provide seamless and continuous interactivity options to users. Different communication systems can be considered to enable wearable multi-system connectivity, such as References [62,63]:

- Public Land Mobile Networks: These range from 2G to future 5G communication systems, which support machine to machine communications (M2M) or purpose specific systems for sensor integration, such as NB-IoT or Cat. Mobile terminals can be used as opportunistic sensor platforms or to provide access gateways in tethered operation to WBAN/WPAN networks.
- WPAN/WBAN: These are wireless communication systems which are intended to support short to medium range communications, have network topologies that are reconfigurable in the face of adverse network conditions (i.e., ad-hoc network configurations), and large scalability. The majority of communication systems have been developed under the scope of IEEE 802.15 standards, such as Bluetooth, ZigBee, RFID/NFC liaison or Ultrawideband (UWB), while there are other alternatives, such as ANT, Zarlink, Sensium, Z-Wave, RuBee, Dash7 and EnOcean, among others.
- WLAN: Wireless local area networks provide multiple functionalities, such as communication gateway capabilities or support for guiding and location systems.

The main advantage in the use of WBAN/WPAN communication systems for the integration of wearable sensor/actuator platforms is the possibility of providing platforms with a small form factor, with efficient energy handling in moderate coverage ranges using low cost transceivers, which can easily be integrated with gateways or tethered to smartphones.

The main challenges these systems must overcome in order to enable their use as wearable devices are the following [64,65,66,67,68,69,70,71]:

- Small form factor and ergonomics: Typically, the circuit footprint is on the order of several mm${}^{2}$ in order to enable their integration with wearable platforms, such as wristbands, watches, earrings, necklaces, or in different types of textiles. Novel approaches have been followed to increase the integration, employing additive manufacturing techniques (ergonomic casings and enclosures, conductive screen printing of electrodes, sensors and antennas), embroidering conductive wires within textiles, or flexible/stretchable electronics.
- Reduced energy consumption: Wearable applications usually have the use of only limited energy sources. Moreover, an extended lifetime of the energy source is a desirable requirement, to increase the user’s comfort in terms of device downtime owing to the need to recharge the device. Energy reduction is provided by hardware (e.g., the use of ultra-low power electronics, optimized circuit routing, etc.) and communication protocol/software (optimized device states and modified MAC protocols, energy efficient routing protocols, cooperative and heterogeneous network schemes). Other aspects, such as restrictions on the antenna size, non-optimal antenna matching conditions, and the influence of the human body on the antenna radiation diagram, also limit the performance and hence affect the available energy. New approaches have been proposed, such as the use of artificial electromagnetic bandgap embedded antennas in order to enhance antenna performance in contact with the human body. Extended device operation can be provided by using energy harvesting schemes (with different options, such as solar energy, thermo-electric conversion such as Peltier cells, piezo-electric transducers or RF/electromagnetic harvesting by using rectennas).
- Seamless communication capabilities: Wearable devices require communication systems which can efficiently transmit and receive the required information, providing optimal coverage/capacity response, with straightforward connection capabilities in order to ease their operation by the users. WBAN/WPAN communication systems, such as Bluetooth (BT)/Bluetooth Low Energy (BLE), RFID or Near Field Communications (NFC) provide simple association procedures, requiring little or no configuration by the user under conventional operating conditions. Moreover, coverage/capacity restrictions must also be taken into consideration, in aspects such as the transceiver density, which can increase the overall interference levels and hence reduce the quality of service parameters. Moreover, user interaction and adoption levels will also be biased by normalization in the use of the technology, in terms of accepting information exchange and ensuring adequate privacy and security levels.

**Table 3:** Summary of RFID and WSN technologies, challenges, and solutions proposed in the state of the art.

Technology	Challenges	Solutions
RFID	Energy harvesting Inflexibility Platform Cost Communication protocols Coverage/Read range	[18,22,37,58] [12,20,23] [20,23,24,37] [22,25] [5,20,25,59] [18,23]
WSN	Latency Reliability Data rate Energy consumption Scalability Communication protocols Security & privacy	[35,36,69] [35,36,56,68,69] [54,68] [10,56,58] [10,70,71] [42,70] [21,40,54,63]
RFID-WSN	Coordination Communication protocols Energy management & Accuracy of sensors	[8,9,59] [5,7] [3,36,58]

**Figure 5:**
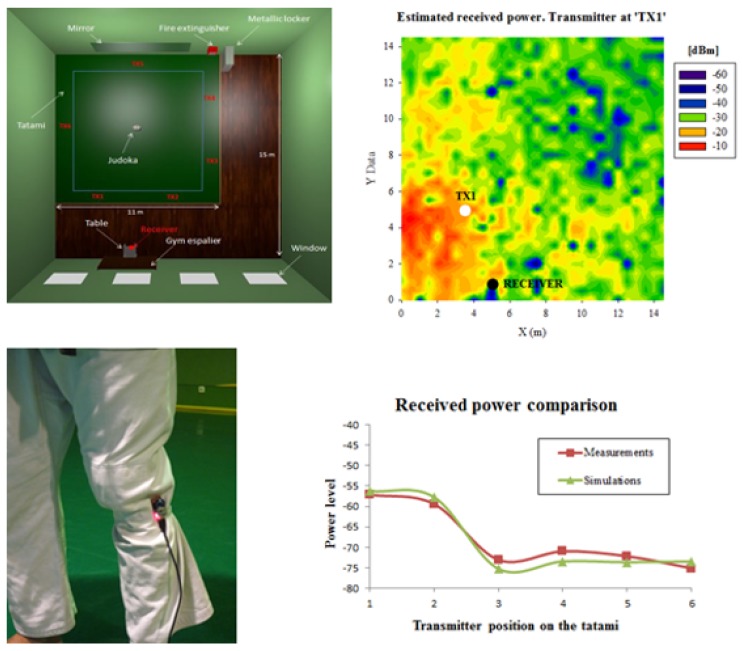
Wearable node integration for judo training/monitoring applications. Device integration has been analyzed in terms of human body impact on node performance in order to optimize the network layout as well as the integration of the device as described in Reference [80].

Due to the features of wearable systems in terms of reduced size, cost and energy consumption, as well as to their enhanced communication capabilities and integration with other devices such as smartphones, their scope of application has become very broad. Hence, wearable technology is being used in leisure/sport activities, health maintenance and monitoring, location/tracking systems for persons and assets, with multiple wearable platforms and different levels of data gathering and analysis [72,73,74]. The main features have been summarized in Table 4. Within the context of healthcare, wearable technology is being developed and tested for different applications, such as the use of head mounted displays (for surgical procedures, support for imaging diagnostics, education and training of medical students or immersive guiding systems), wearable body sensors (monitoring vital signs, posture recognition and recording) or sensorized garments, such as vests, head caps, or pants. Continued research is leading to new applications of wearable technologies in health, such as plantar load monitoring for diagnostics [75] or sleep behaviour analysis [76]. Systems have also been deployed in order to enable data fusion, for example, biomedical signals with user behaviour patterns in order to provide social sensor node applications [77]. Sport and fitness activity is one of the main fields of application of wearable technologies, which has gained increased interest in recent years. The use of wearable technologies finds application in multiple sports (football, soccer, basketball, running, judo, etc.) and with multiple goals, such as training support, injury monitoring and diagnostics, or gathering data about sporting events [78,79,80]. Wearable technology has also been proposed for location and tracking applications of different types. In Reference [81] monitoring and ensuring the security of miner activity, with an integrated system including a smart watch, sensorized helmet, glasses and sensorized vest, is proposed. A system for locating and tele-monitoring domestic pets has been proposed, for indoor/outdoor operation [82]. Further applications have been explored for livestock monitoring, providing multi-system, multi-agent approaches [83]. An example related to sport activities and wearables has been proposed for the application of monitoring training and competition venues within Judo [79], in which system operation has been characterized for the specific conditions of body impact in wireless system operation (see Figure 5).

## 5. Conclusions

IoT is becoming a reality but there are still several challenges that need to be addressed in order for it to be satisfactorily adopted. Two of the main technologies that enable the IoT are RFID and WSN. These technologies, their main applications, and some still unsolved challenges have been reviewed in this paper in order to arouse the inquisitiveness of researchers, so that these two technologies can evolve from research ideas and prototyping to robust and powerful solutions that improve everyone’s life. Some challenges such as poor coverage of RFID readers, the inefficiency of their reading range with passive tags have been mentioned or the limited accuracy of low power sensors in RFID technology. For the case of WSN, the routing protocols and the energy consumption are also to of the vital focuses to be improved.

Two of the main trends identified that will be able to elevate the IoT to the next level are the integration of RFID with WSN in their different modalities, including passive and computational sensors and more robust and less power demanding networks; and wearable sensors, bringing about unobtrusive solutions and applications. However this two proposals are yet to be highly improved.

Some improvement opportunities have been identified. The integration of RFID components with WSN nodes will cause a density increase of components, all of them sharing the same spectrum. This will increase the interference in the communication channel, becoming a research challenge. In addition to this, the communication protocol or the introduction of the multi-hop network feature to extend the range of an RFID-WSN network are shown as opportunities to alleviate this problem, among others. Moreover, RFID-WSN simulators have been detected that can help to develop these systems. 

## Figures and Tables

**Table 1:** Overview of the main areas of application of RFID sensing, including some state of the art examples for each area.

**Health**	**Agriculture**	**Transportation**
continuous patient monitoring inter-departmental communications patients’ mobility Efficient emergency dispatching [18,19,20]	sensor innovations smart irrigation smart tractors monitoring key parameters [21,22]	smart roads environment-connected vehicles people-centric integrated transportation [23,24,25]
**Retail**	**Industry**	**Neuro-Engineering**
security process automation real-time stock control product quality control [26,27]	smart maintenance environment-aware objects real-time monitoring real-time monitoring of the platforms [30,31,32]	biomedical device communications low-power communications backscatter communications [33,34]

**Table 2:** Summary of several Internet of Things (IoT) wireless driving technologies and their main applications in a wireless sensor network (WSN).

Technology	Description and Main Applications
RFID	Radio Frequency Identification Very low power identification, tracking, sensing, indoor positioning
NFC	Near Field Communications Very short range identification, tracking, sensing
BT/BLE	Low Energy Support short to medium range communications. Indoor positioning
2G and 5G	Mobile communications network. Support M2M communications, sensor integration
ZigBee	Communication protocol for WPAN Short distance, low complexity, power, and rate sensor data transmission
UWB	Ultra Wide Band radio techonology Short-range indoor applications. Real-time positioning systems

**Table 4:** Overview of wearable technology and applications in terms of fundamental requirements, enabling technologies and application domains.

Requirements	Enabling Technologies	Application Domains
Small form factor-ergonomics	Additive manufacturing techniques	Sports-Leisure (training, injury monitoring, competition assistance)
Reduced energy consumption	Energy harvesting supercapacitor/batteries-ultra low power consumption	Healthcare (body signal monitoring, ambient assisted living, telemedicine)
Small/moderate coverage-transmission rate	WBAN/WPAN-PLMN-WLAN communication systems	Location and tracking (infants, industrial security, livestock/domestic pet tracking and monitoring)
